# Clinical experience with adjustable scleral lenses

**DOI:** 10.5935/0004-2749.20230009

**Published:** 2023

**Authors:** César Lipener, Juliana Rosa

**Affiliations:** 1 Contact Lens Section, Ophthalmology Department, Escola Paulista de Medicina, Universidade Federal de São Paulo, São Paulo, SP, Brazil

**Keywords:** Contact lenses, Scleral lenses, fitting, Keratoconus, Keratotomy, radial, Refractive surgical procedures, Rehabilitation, Learning curve, Lentes de contato, Adaptação, Ceratocone, Ceratotomia radial, Lente escleral, adaptação de lente escleral, Procedimentos cirúrgicos refrativos, Reabilitação, Curva de aprendizado

## Abstract

**Purpose:**

The aim of this study was to evaluate the fitting process of a scleral lens that allows several parameter adjustments during trials and after the initial period of use. In addition, we verified which adjustments were needed and used the most, their indications, and how often these resources were used, and checked the results.

**Methods:**

Scleral contact lens fittings in a private clinic setting were prospectively analyzed in a sequential, non-randomized, and non-comparative manner. All the patients underwent a complete ophthalmic examination and had an indication for scleral lens use (Zenlens, Alden Optical).

**Results:**

Scleral fit was analyzed in 80 eyes of 45 patients. Regarding diagnosis, 72% of the patients had keratoconus; 12%, radial keratotomy; 5%, post-refractive surgery ectasia; 5%, dry eye; and 3%, high myopia. In 66 (82.5%) of the 80 eyes studied, parameters were modified when the lenses were ordered. The reasons that led to the modifications were apical touch or decreased sagittal height, increased sagittal height, cylindrical over-refraction, poor visual acuity, lens flexure, peripheral touch, 360° edge compression, horizontal edge compression, and vertical edge compression.

**Conclusion:**

In this study, the use of Zenlens scleral lenses was shown to be a promising corrective treatment for patients requiring the use of scleral lenses. Although the study suggests a learning curve, as many adjustments were allowed, the lens could be customized according to the patients’ needs. This increased the success rates of fitting and wearing, and consequently, use of the lens became a great option for the visual rehabilitation of patients.

## Abstract (pt)

**Objetivo:**

Avaliar o processo de adaptação de uma lente escleral que permite vários ajustes de parâmetros durante os testes e após o período inicial do seu uso; verificar quais os ajustes foram necessários, quais foram os mais utilizados, as suas indicações, a frequência com que estes recursos foram utilizados, e avaliar os resultados das mudanças realizadas.

**Métodos:**

A adaptação da lente de contato escleral foi analisada prospectivamente, de forma sequencial, não aleatória e não comparativa. Todos os pacientes foram submetidos a um exame oftalmológico completo e tinham indicação para o uso de lentes esclerais. Foi utilizada a lente Zenlens (Alden Optical).

**Resultados:**

Foi analisada a adaptação de lentes de contato esclerais em 80 olhos de 45 pacientes. Quanto ao diagnóstico, 72% tinham ceratocone, 12% tinham sido submetidos a ceratotomia radial, 5% tinham ectasia pós-cirurgia refrativa, 5% tinham olho seco, e 3%, alta miopia. Em 66 dos 80 olhos estudados (82,5%), os parâmetros foram modificados quando as lentes foram encomendadas. As razões foram: toque apical ou diminuição da altura sagital, aumento da altura sagital, sobre-refração cilíndrica, baixa acuidade visual, flexão da lente, toque periférico, compressão da borda em 360° e compressão da borda horizontal e/ou vertical.

**Conclusão:**

O uso de lentes esclerais Zenlens demonstrou ser uma forma de correção muito promissora para os pacientes que requerem o uso de lentes esclerais. Embora o estudo sugira uma curva de aprendizagem, é possível personalizar as lentes de acordo com as necessidades de cada pacientes. Este fato melhora a adaptação e aumenta a chance de sucesso do uso.

## INTRODUCTION

Scleral lenses have undoubtedly become a real option in the field of modern contact lenses, expanded the possibility of visual rehabilitation for patients with irregular corneas, and provided a good resource for subjects who present dry eye and other ocular surface alterations.

Scleral lenses were the first contact lenses developed and described by Adolf Fick in 1888^(1)^, who had the idea of correcting corneal irregularities with glass scleral shells that would increase patients’ visual acuity^(2)^. The use of scleral lenses became popular with the advent of polymethylmethacrylate (PMMA) in 1936 and in 1938, when Obre and Muller made the first lens with this material^(3,4,5)^. However, these lenses were progressively replaced by corneal lenses. The interest in these was renewed when scleral lenses started to be made with new gas-permeable materials.

The development of new designs and even better new materials with higher oxygen permeability in the last decade has a direct relationship with the wi despread use of this kind of correction. This allowed many patients, previously with no options, to have a successful fit and vision improvement with these lenses.

The use of scleral lenses made of materials with high oxygen permeability (Dk) reduced the incidence of complications related to hypoxia when compared with PMMA lenses, creating new indications and potential uses for this kind of lens. Some authors started to classify these lenses according to diameter (full, large, or miniscle ral) and, consequently, in which region they would lean on, but all of them share the same principle; that is, they reach beyond the limbus. Fitting scleral lenses with no corneal contact offers some advantages as follows:

- More stability of parameters for high-power lenses;
- Greater chance of fitting success in corneas with irregular topographies;
- Less palpebral sensation and greater stability, with increasing comfort;
- Lower risk of foreign bodies being trapped under the lens;
- Lower risk of losing the lens due to palpebral action;
- Presence of a liquid reservoir between the cornea and the lens: In addition to improving dry eye symptoms, this optically neutralizes most of the irregular astigmatism and protects the corneal surface. This can also be useful in chronic epithelial defects and other ocular surface conditions.

Despite these advantages, some characteristics of this contact lens may discourage its use, such as the following:

- Expensive manufacturing when compared with other types of lens;
- Fitting requires new skills and has a learning curve;
- Its diameter/size may intimidate some patients, causing a volume sensation and/or a pseudo-proptosis appearance, mainly in monocular fittings;
- Lens insertion requires a relative motor skill, with adequate head position and precise hand movements to prevent leakage of the fluid chamber or air bubbles trapped under the lens.

Hypoxia is a major concern mainly in some situations such as in post-keratoplasty fitting; may occur in some cases, mainly in lenses made of low-Dk materials, which are rare nowadays; and may be linked to excessive vaulting and/or high-thickness lenses^(6,7,8,9,10,11,12,13,14)^. Thus, scleral lenses are optical and therapeutic devices that may be an excellent option when soft or rigid gas-permeable (RGP) corneal lenses cannot be successfully fit.

Therefore, the main indications are as follows: soft or RGP corneal lens intolerance, inadequate lens-cornea relationship, excessive mobility and/or instability, or insufficient visual acuity improvement. In these cases, before the advent of scleral lenses, patients had no other option but to undergo a surgical procedure.

Many scleral lenses by different manufacturers are available in other markets, but only few are available in Brazil. They all have their own design and parameters, based on which manufacturers claim that their lenses have advantages and are better than the competitors’ products. The greater the possibility to have a customized fitting based on individual ocular findings, patient’s topography, and associated pathologies, the higher the chance of success.

Despite the current trend of scleral lens fitting customization, when this study was started, not all lenses allowed modifications to be made in their parameters to improve fitting. The launch of Zenlens (Alden Optical/ B&L), which started to be manufactured in the Brazilian market in 2015 and allows for parameter changes, kindled an interest in evaluating the efficacy of these modifications.

## METHODS

Scleral contact lens fittings in a private clinical setting were analyzed in a sequential, non-randomized, and non-comparative manner. All the patients underwent a complete ophthalmic examination and had an indication for contact lens use.

The inclusion criterion was the need for scleral lens when other contact lens fitting was impossible or suboptimal owing to discomfort or excessive instability or mobility. The exclusion criteria were glaucoma, active inflammatory or infectious conditions, corneal hypoes-thesia, inability to handle the lens, and pregnancy. The lens used in this study was Zenlens (Alden Optics), manufactured in Brazil by Solótica. All the patients signed an informed consent form and were informed and enlightened about the study and the possible consequences of using the lens.

### Contact lens

#### Zenlens (Alden Optical - B&L, made in Brazil by Solótica)

This a scleral contact lens with an asymmetrical multicurve design that allows for customized adjustments in the anterior and posterior surfaces of the lens in an orderly and standardized manner. The modifications that can be made include the anterior toricity, limbal clearance curve (LCC) adjustment, flexure control, advan ced peripheral system (APS), and microvault.

All these parameter modifications when ordered separately are made without changing the sagittal height (SAG) and other parameters due to the special region of the lens named SmartCurve (SmartCurve Technology). The trial set has 24 lenses, 12 for prolate corneas and 12 for oblate corneas, each subset consisting of six each of 16- and 17-mm-diameter lenses.

### Anterior toricity

Allows correction of all forms of residual astigmatism (corneal, refractive, hypercorrection, or hypocorrection) and can lead to visual acuity improvement.

### LCC adjustment

LCC allows increased limbal vault of up to 150 µm, and it is an interesting option when a touch point exists or the vault is reduced in this area. The LCC may also be adjusted when despite a normal vault in this area, one plans to order landing zone flattening, which will probably reduce the vault in the region.

### Flexure control (or flexibility ring)

In some cases, pressure and compression on the lens surface may cause flexure of the lens at its optic zone, which may appear as a cylindrical over-refraction, unexpected spherical over-refraction, or poor quality of vision. This can be observed on topography imaging, shown as symmetrical or asymmetrical astigmatic images. In these cases, structural strengthening around the optic zone increases resistance in this area, preventing flexure, eliminating or reducing cylindrical over-refraction, and resulting in better visual acuity.

### Advanced peripheral system

APS allows independent lowering or lifting of the horizontal and vertical meridians of the landing zone (in 10 steps of 30 microns each). When asymmetrical, it may cause toricity of the lens, but when done equally in both meridians, it promotes lowering or lifting at the peripheral portion by 360°.

When this modification is needed, the first information to be communicated is whether the modification will be made in 360° of the lens or in just one meridian (vertical or horizontal) or both. Different changes (lowering in one and lifting in the other) in different degrees are not possible. When the change is to be made in the whole periphery, we ask for APS Flat for lifting and APS Steep for lowering, and this can be performed in 30-µm increments.

To achieve this in just one meridian, APS Steep or Flat, vertical or horizontal, can ordered and information on how much change is necessary can be obtained, such as APS horizontal Flat 2 or APS vertical Steep 3. It may also be achieved in both at the same time, for example, APS horizontal Flat 2 and APS vertical Steep 3.

### Microvault

This technology allows for the lifting of a sector of the lens. To accomplish this at the right position and with adequate extension and lift, the manufacturer should be informed about the axis (in 10° steps), distance from the edge (in mm), width (in mm), and the intended lifting (up to 500 µm).

### Lens profile, diameter, and fitting routine

The lens has two designs, one for oblate corneas (post-refractive surgery and keratoplasty) and the other for prolate corneas (ectasias). It also offers two-diameter options, 16 mm (for corneas with diameters <11.7 mm) and 17 mm (for corneas with diameters >11.8 mm).

The fitting was started following the manufacturer’s instructions regarding profile, diameter, and first trial lens. The lens was inserted with its concavity filled with sterile 0.9% saline solution with no preservatives plus one drop of 1% sodic fluorescein (Allergan).

The first evaluation was performed after a minimum period of 30 minutes and involved checking the centralization, compression or lifting of the edge in four quadrants (nasal, temporal, superior, and inferior), and estimating the sagittal depth, whose adequate value was considered to be 250 µm.

During the fitting trials, besides the parameters modified using the trial set lenses (diameter, oblate or prolate profiles, sagittal height, and base curve), other specific adjustments were requested for some patients. For some of patients, these modifications were requested not only when final lenses were ordered but also after a variable period of wearing, according to each patient’s needs.

After the trials, a spherical cylindrical over-refraction was attained, and the final order was placed with all the modifications and adjustments needed to improve the fit. In some patients, a new topography imaging over the contact lens was performed to observe the presence of astigmatism patterns, which are suggestive of lens flexure.

When the patients returned to get their lenses, if the visual acuity and lens fitting were according to the trials, they were instructed about lens insertion, removal, and maintenance, and a new examination was scheduled in 15 days. In case of any visual complaint related to over-refraction or other signs or symptoms (hyperemia, discomfort, handling, blurring, etc.), after a new evaluation of the lens, new lenses were ordered with the modifications deemed necessary to solve vision and/or fitting complaints.

## RESULTS

### Patients

Contact lens fitting was evaluated in 80 eyes of 45 patients, including 22 females (48.89%) and 23 males (51.11%).

Regarding diagnosis, 72% of the patients had keratoconus; 12%, radial keratotomy; 5%, post-refractive surgery ectasia; 5%, dry eye; and 3%, high myopia.

Among the patients with keratoconus (58 eyes), 8 (13.79%) underwent corneal crosslinking (CXL); 8 (13.79%), corneal ring implantation; and 8 (13.79%), keratoplasty. Table 1 shows the reasons that led to an indication of scleral lens:

**Table 1.** Scleral lens indication

Intolerance	40%
Poor fitting	30%
Poor visual acuity	10%
Already using scleral lenses	10%
Dry eye	8%
Residual astigmatism	2%

When scleral lenses were indicated for patients with keratoconus who had not received a ring implant or undergone a keratoplasty performed, 42 eyes had keratoconus (no ring implant or keratoplasty), 17 eyes were fitted for lenses because of intolerance for corneal RGP lenses (41.46%), 14 had poor fitting of corneal RGP lenses (34.14%), 6 had poor visual acuity with other lenses (14.64%), and 4 already were scleral lens users (9.76%)

As regards to corrected visual acuity (with glasses), the following distribution was observed: in 14 eyes (17.50%) refraction was impossible; in 10 (12.5%), it was =0.1; in 12 (15%), it was between 0.1 and 0.25, in 37 (46.25%), it was >0.25 and <0.50; and in 7 (8.75%), it was =0.50

### Lens adjustments

Table 2 shows the number of trial lenses tested before final order.

**Table 2.** Trials performed before ordering the lenses

1 trial - 21 eyes	26.25%
2 trials - 42 eyes	52.5%
3 trials - 13 eyes	16.25%
4 trials or more - 4 eyes	5%

When only the 41 eyes with keratoconus without a prior procedure (keratoplasty or ring implant) were included in the analysis, we performed 1 trial in 11 eyes (26.82%), 2 trials in 22 eyes (53.65%), and 3 trials in 8 eyes (19.51%).

In 66 of the 80 eyes included in the study, parameter modifications were requested when the lens were ordered for the following reasons: apical touch or decreased sagittal height, increased sagittal height, cylindrical over-refraction, poor vision acuity, lens flexure, peripheral touch, 360° edge compression, horizontal edge compression, and vertical edge compression.

In some cases, more than one modification was necessary, which means that the modifications that could be ordered were not mutually exclusive. The modifications and their frequencies were as follows: toric APS for 35 eyes (43.75%), SAG modification for 26 eyes (32.5%), anterior toricity for 20 eyes (25%), flexure control for 15 eyes (18.75%), thickness increase for 8 eyes (10%), LCC adjustment for 5 eyes (6.25%), total APS for 8 eyes (1%), and no change for 14 eyes (17.5%).

When only the patients with keratoconus who had no history of previous keratoplasty or ring implant (41 eyes) were included in the analysis, the distribution of the modifications ordered initially were as follows: toric APS for 20 eyes (48.78%), SAG modification for 18 eyes (43.9%), anterior toricity for 7 eyes (17.07%), flexion control for 11 eyes (26.82%), thickness increase for 4 eyes (9.75%), LCC adjustment for 2 eyes (4.87%), total APS for 1 eye (2.43%), and no change for 6 eyes (14.63%).

Regarding APS modifications, we found that 43 eyes needed APS modification; for horizontal flat: 39 eyes (90.69%) needed Flat 1 in 14 lenses and Flat 2 in 25 lenses; and for vertical flat: 19 eyes (44.18%) needed Flat 1 in 16 lenses and Flat 2 in 3 lenses; for V steep: 14 eyes needed Steep 1 in 8 lenses and Steep 2 in 6; for V standard: 10 eyes; and for H standard: 4 eyes. No order for horizontal steep was necessary in any case.

Regarding the 43 lenses in which APS was modified, in 28 lenses (65%), no further APS modifications were needed after the lenses were delivered. Among those that required other modifications, 15 and 19 had modifications for a steeper and flatter meridian, respectively. When analyzing the APS modifications only in the patients with keratoconus, we found the following distribution: APS modification was made in 20 eyes; horizontal flat: 18 eyes (90%), Flat 1 in 6 and Flat 2 in 12; horizontal steep: not ordered in any of the cases; vertical flat: 6 eyes (30%); and vertical steep: 7 eyes (35%).

Another interesting information that we gained from this study was the number of necessary modifications until good fit and visual acuity were reached, including those made in the initial order (Table 3).

**Table 3.** Number of modifications made

Modifications made	Number of eyes	%
None	6	7.5
1	33	41.25
2	25	31.25
3	10	12.50
4	4	5
5	2	2.5

In 14 lenses, no parameter modifications were necessary at the initial order; however, one or more fitting patterns or visual acuity differences were found during the dispensing exam as follows: 2 with increased SAG, 1 with decreased SAG, 1 with apical touch, 3 with decreased visual acuity with some over-refraction, and 4 with sector edge compression. In 5 eyes, the lenses were well fitted, and vision was compatible with its state in the final trial.

Sagittal height (SAG) was considered high in 36 eyes. In 13 cases, the lens was not changed at that time and reevaluation was performed after 15 days of lens wearing. In 23 eyes, a SAG decrease was ordered for 11 eyes (47.82%) because of SAG improvement. In 10 eyes (43.47%), the SAG continued to increase, and in 2 eyes (8.69%), the SAG seemed to decrease more than expected.

In 29 eyes, a decreased SAG was observed during examination. In these eyes, the lens was not changed and reevaluation was performed in 15 days in 6 eyes (20.68%). In 23 lenses (79.32%), a new lens was ordered with greater SAG due to improved SAG in 15 eyes (65.2%), persistent decrease in SAG in 5 (21.73%), and suspected increase in SAG in 3 (13.04%).

To correct residual astigmatism, anterior toricity was used in 20 lenses, of which 14 (70%) required no other modification and 6 (30%) had another adjustment in the final lens power. In some cases, a poorer visual acuity than expected was possibly associated with lens flexibility. For these cases, to improve visual acuity, a flexure ring was ordered. Of the 15 lenses in which this resource was used, 10 (66.7%) showed improved vision and 5 (33.3%) showed no change. In 8 cases, an increased central thickness, from 350 µm to 450 µm, was requested to improve visual acuity. In 4 patients, visual acuity improved, while in the remaining 4, no change was observed.

Limbal lifting adjustment was necessary in 5 lenses because of touching at the limbal area, of which 3 showed improvement and 2 required another increase to be ordered. As expected, visual acuity improved with scleral lenses in a significant number of eyes, as shown in tables 4 (visual acuity with glasses) and 5 (visual acuity with scleral lens).

**Table 4.** Improvement of visual acuity with glasses

Impossible to measure	14
Equal or <0.1	10
>0.1-0.25	12
>0.25-0.5	37
>0.5-1.0	7

**Table 5.** Improvement of visual acuity with scleral lenses

Impossible to measure	1
Equal or <0.1	1
>0.1 -0.25	4
>0.25 - 0.5	28
>0.5 - 1.0	46

In 1 patient, visual acuity could not be measured because of bullous keratopathy. Visual acuity with glasses was =0.1 in 12.5% of the eyes, while with scleral lenses, the VA remained in this range in just 1.25% of cases.

The proportion of eyes with visual acuities between 0.1 and 0.25 decreased from 15% to 5%, and the proportion of those with visual acuities ranging from 0.25 to 0.5 decreased from 46.25% to 35%. The proportion of cases with better visual acuity between 0.50 and 1.0 corresponded to 8.75% with correction, which increased to 57.5% with scleral lenses.

**Figure 1.**
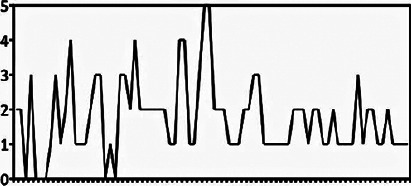
Numbers of parameter modifications over time, showing that in the last 30 eyes studied, only 1 or 2 modifications were necessary (including those of the initial order) in 29 eyes and 3 changes were ordered in just one eye.

Regarding complications, the following were observed in 4 eyes, the patients complained of discomfort after wearing the lens for just 4 hours a day. One patient stopped wearing the lenses because of keratoplasty failure and bullous keratopathy. In another patient, a neovascularization in the RK incision was detected. In 2 eyes, perilimbal hyperemia was observed, with edema, folds, and keratitis, and the patient did not return after a new orientation. One patient stopped wearing the lens after developing hydrops, and 1 patient had corneal edema despite a well-fitted lens. Another observation during the study was the decreasing number of modifications over time, as shown in figure 1.

## DISCUSSION

As we previously mentioned, scleral lenses have undoubtedly become a real option in the field of modern contact lenses and continue to evolve because of the great advances in manufacturing technology. One of their distinguishing points is the possibility to customize the lens, with alternatives to change design, edge lift, sagittal height, and quadrant modifications, allowing correction of problems that previously could lead to events or complications that could compromise lens wearing.

A scleral lens covers the cornea and limbus and “lands” over the bulbar conjunctiva, which overlays the sclera. Thus, the lens should have a sagittal height greater than the cornea in all its dimensions. The corneal sagittal height complexity is influenced by corneal elevation and eccentricity, which is, in turn, related to lens diameter and the central and peripheral curvature radii^(15)^.

Considering the lens used in this study, owing to a region of the lens named SmartCurve, some parameters can be modified, such as sagittal height, without the need to modify other parameters.

Various factors are involved in fitting these lenses, many of which are related to the anatomical characteristics of the limbic area and sclera. Several attempts have been made to obtain objective measures to make the fitting process easier and better. Weber et al.^(14)^ estimated the sagittal height of a scleral lens by using measurements from Pentacam, such as corneal astigmatism and sagittal height. This is, however, an expensive approach, and despite its usefulness, only a few practitioners would have this resource available routinely.

Van de Worp^(16)^ emphasizes the importance of the limbus profile and scleral angle, highlighting that these vary greatly among the population. This study, which was conducted at Pacific University, measured a tangential corneoscleral angle at the horizontal meridian and showed that in most cases, the nasal portion is flatter than the other portions, which is coincident with the topographic finding that shows greater peripheral flattening of the corneal nasal quadrant. These findings may explain the necessity for a toric periphery in many cases, justifying the need to flatten the horizontal meridian in 90% of the cases.

According to Barnett and Fadel^(15)^, larger lenses may benefit from toric landing zones to decrease the possibility of complications, including decentration, lens distortion, air bubbles, blanching, conjunctival prolapse, and fogging. Besides, larger lenses can improve comfort, increase wearing hours, and benefit optical correction. When a spherical lens is fitted over a toric sclera, the lens will touch the conjunctiva over the scleral flat meridian and stay farther away from the steeper meridian. It is more difficult when this mismatch occurs in just one quadrant, requiring a lens that offers adjustment by quadrant, which will soon be available in our market.

The possibility of doing spherical and cylindrical corrections due to an anterior toric surface has also proven to be an important resource, which was used in 25% of cases and may be considered as another distinguishing aspect of this kind of lens among lenses with corneal designs for irregular corneas, most of which do not offer this possibility.

Another parameter modification was the flexure ring, used in cases with evidence of lens flexure when performing topography over the lens or even when visual acuity fell below expectations, as the ring improves flexure resistance. In situations where the ring cannot be used, to achieve the same goal, the lens central thickness can also be increased, but this modification may compromise oxygen transmission.

Regarding the indications of scleral lenses, the literature reports 80.3% of optical indications and 16.8% of therapeutic indications^(6)^.

The main optical indications are primary corneal ectasia (keratoconus, keratoglobus, and pellucid marginal degeneration), ectasia and/or irregular astigmatism (secondary to keratoplasty, refractive surgery, or post- trauma corneal irregularities), aphakic eyes, and high myopia^(11)^. Patients with this kind of indication, that is, aiming to improve visual acuity, benefit greatly from scleral lenses owing to the uniform and stable lacrimal film between the eye and posterior surface of the lens, which can correct optical defects related to irregular astigmatism, even with lenses with high refractive powers^(1)^.

As regard to the therapeutic indications of scleral lens, the main conditions are scarring diseases of the cornea and conjunctiva (Stevens-Johnson syndrome, ocular cicatricial pemphigoid, cicatricial entropion, and post herpetic keratitis) and cases of severe dry eye (exposure keratopathy, congenital deficiency of the meibomian glands, superior limbic keratoconjunctivitis, and Sjögren syndrome)^(8,9,10,11,12,13,14,15,16)^. This kind of use is related to lacrimal retention between the cornea and lens and ocular protection in cases of exposure keratitis and eyelid or eyelash abnormalities.

In our sample, 72% had keratoconus, 12% underwent radial keratotomy, 5% had post-refractive surgery ectasia, 5% had dry eye, and 3% had high myopia. In other words, in this sample, the number of patients with therapeutic indications was a little lower than those in other studies.

Finally, as shown in figure 1, another interesting point that could be observed was that the number of modifications of the lens parameters decreased over time, which suggests the existence of a learning curve. As the examiner gets more experience fitting these lenses, the number of modifications required tends to decrease.

The use of Zenlens scleral lenses was shown to be a promising corrective treatment for patients with an indication of scleral lenses. Although the study suggests a learning curve, many parameter adjustments are possible, which allows for the customization of the lens according to each patient’s needs. This targeted approach increases the success rates of fitting and wearing, making this a great option for the visual rehabilitation for these patients.
